# Risk Factors of Gestational Diabetes Mellitus Among Women Attending an Antenatal Care Clinic in Prince Sultan Military Medical City (PSMMC), Riyadh, Kingdom of Saudi Arabia: A Case-Control Study

**DOI:** 10.7759/cureus.44200

**Published:** 2023-08-27

**Authors:** Maha M Alduayji, Mohie Selim

**Affiliations:** 1 Preventive Medicine Division, Family and Community Medicine Administration, Prince Sultan Military Medical City (PSMMC), Riyadh, SAU; 2 Department of Public Health and Community Medicine, Faculty of Medicine, Assiut University, Assiut, EGY

**Keywords:** case-control study, antenatal care clinics, saudi arabia, risk factors, gestational diabetes mellitus

## Abstract

Background: Gestational diabetes mellitus (GDM) is a serious health issue for both mother and child. As GDM is common worldwide, healthcare providers pay attention while screening and managing pregnant women to ensure good outcomes for both mother and child.

Objective: This study aims to identify the risk factors associated with developing GDM in pregnant women attending antenatal care clinics in Prince Sultan Military Medical City (PSMMC) in Riyadh, Saudi Arabia.

Methods: This is a case-control study that utilized patients' medical records for data collection. The study included 317 pregnant Saudi women who attended antenatal care clinics and antenatal diabetic clinics in PSMMC from May 2022 to May 2023. Cases were defined as women who met the inclusion and exclusion criteria and had a positive oral glucose tolerance test (OGTT) result, while controls were defined as women in the same age group and gravidity who had negative OGTT. Analysis was conducted using SPSS Statistics version 29.0 (IBM Corp. Released 2021. IBM SPSS Statistics for Windows, Version 29.0. Armonk, NY: IBM Corp.)

Results: The total number of cases was 132 out of 313 total samples, representing 42.2% of the total sample. Three factors were associated with an increased risk of developing GDM, including a family history of diabetes (p-value <0.001), a history of GDM (p-value <0.001), and macrosomia (p-value = 0.020). The study also found higher BMI and advanced maternal age were risk factors for GDM (p-value = 0.004, 0.007), respectively. However, the study did not find a significant association between GDM and other factors, such as chronic disease prevalence, history of miscarriage, or history of fetal death.

Conclusion: The study identified several risk factors associated with an increased risk of GDM including family history of diabetes, history of GDM, macrosomia, overweight/obesity, and advanced maternal age. It is recommended that antenatal care providers screen for GDM risk factors and closely monitor overweight, obese, or older women. Education and counseling on healthy lifestyle habits, such as maintaining a healthy weight and engaging in physical activity, may also be beneficial for preventing GDM. Further research is needed to confirm and identify additional risk factors for GDM.

## Introduction

Gestational diabetes mellitus (GDM) is a type of diabetes mellitus (DM) that is defined as a different degree of carbohydrate intolerance that first manifests during pregnancy or begins during early pregnancy [1]. Diabetes during pregnancy negatively impacts maternal and prenatal outcomes [2]. Hormonal changes during pregnancy that affect insulin production and utilization are the major cause of GDM [3]. GDM is a serious health concern for both mother and child, with potential complications including pre-eclampsia, preterm labor, and macrosomia. Additionally, women with a history of GDM have an increased risk of developing type 2 DM (T2DM) later in life [4]. GDM has become increasingly prevalent worldwide [5]. It is estimated that 13.9% of pregnancies worldwide are affected by GDM, while the United States experiences GDM in 2% to 10% of all pregnancies per year [1,6]. In Saudi Arabia, the prevalence of GDM is estimated at 15.5% [7].

Several well-known risk factors have been reported as predictors of the progression of DM during pregnancy. These factors include obesity, physical inactivity [8], advanced maternal age [9], multiparity, family history of T2DM, and certain ethnicities, including Asians [10], a previous macrocosmic child, GDM in the previous pregnancy [11], and polycystic ovarian syndrome (PCOS) [12]. As reported in a recent meta-analysis, overweight/obesity, hypothyroidism, PCOS, and a family history of DM qualify as convincing or highly suggestive risk factors for GDM [13]. GDM is diagnosed via the oral glucose tolerance test (OGTT), which is recommended for pregnant women at high risk of GDM during early perinatal visits and all pregnant women between weeks 24 and 28 of pregnancy [11]. To diagnose GDM, the patient must have one or more of the following: fasting plasma glucose level ≥5.1 mmol/L (92 mg/dL), one-hour postprandial plasma glucose ≥10.0 mmol/L (180 mg/dL), or two-hour postprandial plasma glucose ≥8.5 mmol/L (153 mg/dL) [10].

GDM is a serious health issue for both mother and child. It can cause pre-eclampsia, preterm labor, and macrosomia. Women with a history of GDM are also at risk of developing T2DM later in life. As GDM is common worldwide, healthcare providers should be careful with screening and management to ensure good outcomes for both mother and child. Our study aimed to identify risk factors associated with developing GDM in pregnant women attending antenatal care clinics in Prince Sultan Military Medical City in Riyadh (PSMMC), Saudi Arabia.

## Materials and methods

Study design

This is a case-control, record-based study.

Study area

The study took place in PSMMC in Riyadh, Saudi Arabia.

Study population

The study population consisted of pregnant Saudi women aged 20-45 years who attended antenatal care clinics and antenatal diabetic clinics in PSMMC from May 2022 to May 2023. Cases and controls were selected from antenatal clinics in the Al-Wizarat Healthcare Centers, a branch of PSMMC.

Inclusion and exclusion criteria

The inclusion criteria for this study were Saudi women between the ages of 20 and 45 who had a documented OGTT during pregnancy between May 2022 and May 2023. Exclusion criteria included pre-pregnancy diabetes (type 1 or type 2), as well as pregnant women with an estimated delivery date after May 2023.

Cases were defined as women who met the inclusion and exclusion criteria and had a positive OGTT test result. Controls were defined by their negative OGTT results.

Selection of case group and control group

The total sample size was estimated to be 375 using the epi-info website [14]. To achieve 80% power and a 95% confidence level, the case-control ratio was set to 1:2. Thus, our sample was divided into 125 cases and 250 controls.

We obtained lists of patients who visited an antenatal care clinic and an antenatal diabetic clinic during their pregnancy from the hospital's registries after obtaining the necessary permissions. Control selection was based on age group and gravidity to match the cases.

Data collection

Data from the files of the selected patients were collected using a prepared data collection form.

The study's variables can be categorized into three main sections: demographic information (e.g., age, weight), pregnancy-related information (e.g., gravidity, gestational age, and history of GDM), and medical information (e.g., chronic diseases and fasting plasma glucose).

Diagnostic criteria

In the current study, the diagnosis of GDM was made according to the new criteria established by the International Association of Diabetes and Pregnancy Study Groups (IADPSG) [15]. All women between 24 and 28 weeks of gestation underwent evaluation with the 75-g OGTT. The diagnosis of GDM was considered if one or more of the following criteria were present: fasting glucose ≥5.1 mmol/L, one-hour glucose ≥10 mmol/L, or two-hour glucose ≥8.5 mmol/L.

Statistical analysis

SPSS Statistics version 29.0 (IBM Corp. Released 2021. IBM SPSS Statistics for Windows, Version 29.0. Armonk, NY: IBM Corp.) was used for data analysis. Descriptive statistics were used to summarize the study population's demographic and clinical characteristics. To test for normal distribution, we used the Shapiro-Wilk test. The results showed that all the variables were skewed, which is why we used the median and IQR to summarize continuous variables. Frequencies and percentages were calculated for categorical variables. The case and control groups were compared using the chi-square test or Fisher's exact test for categorical variables and the Mann-Whitney U test for continuous variables. A p-value less than 0.05 was considered statistically significant.

Ethical consideration

As this is a case-control study, no explicit consent was obtained from participants. However, the study obtained ethical approval from the PSMMC Institutional Review Board (E-2089) and ensured that the rights, safety, and well-being of the participants were protected. The medical records of the participants were accessed only for research purposes and were kept confidential. The study did not offer any incentives or compensation to the participants.

## Results

The study included 313 participants. The age ranged from 20 to 45 with a median of 31 years and an IQR of 28-35 years. The median BMI was 28 (IQR 24-32). The median gestational age at the time of recruitment was 26 weeks (IQR of 25-27 weeks). Cases had a significantly higher median age (32.5 vs. 30 years) and median BMI (28.5 vs. 27) compared to the control group. Demography results of the total sample with a comparison between cases and controls are shown in Table 1.

**Table 1:** Demographic results of the total sample N: number of participants, IQR: interquartile range, BMI: body mass index

N = 317	Total sample	Case		Control		
	Median (IQR)	Median	IQR	Median	IQR	p-value
Age	31 (28-35)	32.5	28-36.5	30	27-34	0.007
BMI	28 (24-32)	28.5	26-33.5	27	23-32	0.004

Of the participants, 42.2% were diagnosed with GDM based on plasma glucose levels and glucose tolerance tests. The median fasting glucose level was 4.4 mmol/L with an IQR of 4.2-4.8 mmol/L. The median one-hour glucose level was 4.5 mmol/L with an IQR of 7-9.8 mmol/L. However, the median two-hour glucose level was 7.3 mmol/L (IQR 6-8.7 mmol/L). Similarly, the median hemoglobin level was found to be 11.3 g/dL (IQR of 10.6-12.1 g/dL).

Our analysis did not reveal significant differences in smoking status or chronic disease prevalence between the two groups. The demography, smoking status before pregnancy, and the presence of chronic diseases were compared between cases and controls in Table 2.

**Table 2:** Comparison of smoking status before or during pregnancy and the presence of chronic diseases between cases and control N: number of participants

N = 317			Case		Control		p-value
		N (%)	N	%	N	%	
Smoking	Yes	3 (1%)	3	100%	0	0%	0.074
	No	310 (99%)	130	41.4%	184	58.6%	
Chronic diseases	Yes	98 (31.3%)	47	47.5%	52	52.5%	0.176
	No	215 (68.7%)	86	39.4%	132	60.6%	

The past medical history showed the following percentages for each factor, from highest to lowest: history of fetal death (29.4%), history of miscarriage (28.1%), and history of GDM (9.9%). Further results are shown in Table 3.

**Table 3:** Past medical history among the included pregnant women GDM: gestational diabetes mellitus

	Yes (%)	No (%)
GDM diagnosis	132 (42.2%)	181 (57.8%)
History of GDM	31 (9.9%)	282 (90.1%)
Macrosomia	10 (3.2%)	301 (96.8%)
History of miscarriage (n=311)	88 (28.1%)	225 (71.9%)
History of fetal death	92 (29.4%)	221 (70.6%)
History of congenital anomalies	17 (5.4%)	296 (94.6%)
Multiple gestations	1 (0.3%)	312 (99.7%)

**Figure 1:**
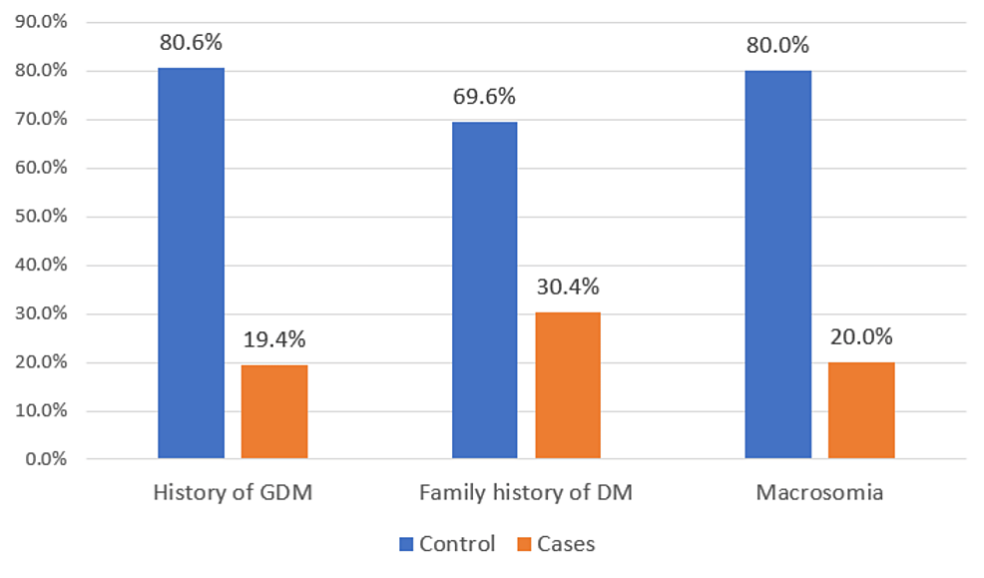
A comparison of the prevalence of GDM among patients who have statistically significant risk factors (p-value <0.05) GDM: gestational diabetes mellitus, DM: diabetes mellitus

The association between GDM and past medical history was analyzed to determine significant associations. The following factors were significantly associated with GDM: history of GDM (p-value <0.001), macrosomia (p-value =0.016), and family history of DM (p-value <0.001). Significant findings are demonstrated in Figure 1.

There was no significant association between GDM and a history of miscarriage, history of fetal death, history of congenital anomalies, multiple gestation, corticosteroid use before pregnancy, or chronic disease prevalence. All the results of the association are shown in Table 4.

**Table 4:** Comparison of the risk factors between cases of GDM and controls *p-value calculated using chi-square test, **p-value calculated using Fisher's exact test, GDM: gestational diabetes mellitus, DM: diabetes mellitus

		Classification		
		Case (%)	Control (%)	p-value
History of GDM	Yes	25 (80.6%)	6 (19.4%)	<0.001*
	No	107 (37.9%)	175 (62.1%)	
Macrosomia	Yes	8 (80%)	2 (20%)	0.020**
	No	123 (40.9%)	178 (59.1%)	
History of miscarriage	Yes	42 (47.7%)	46 (52.3%)	0.252*
	No	90 (40%)	135 (60%)	
History of fetal death	Yes	41 (44.6%)	51 (55.4%)	0.616*
	No	91 (41.2%)	130 (58.8%)	
History of congenital anomalies	Yes	7 (41.2%)	10 (58.8%)	1.000*
	No	125 (42.2%)	171 (57.8%)	
Multiple gestations	Yes	0 (0%)	1 (100%)	1.000**
	No	132 (42.3%)	180 (57.7%)	
Corticosteroids use before pregnancy	Yes	4 (50%)	4 (50%)	0.725**
	No	128 (42%)	177 (58%)	
Family history of DM	Yes	39 (69.6%)	17 (30.4%)	<0.001*
	No	92 (36.9%)	157 (63.1%)	

## Discussion

Our study included 313 pregnant women with the purpose of investigating risk factors for developing GDM. The results showed that various factors were linked to an increased risk of GDM, such as a family history of diabetes (p-value <0.001), a history of GDM (p-value <0.001), and macrosomia (p-value = 0.020). However, the study did not find a significant association between GDM and other factors, such as chronic disease prevalence, history of miscarriage, or history of fetal death. These findings suggest that antenatal care providers should screen pregnant women for GDM risk factors, including family history and previous GDM. Close monitoring is necessary for overweight, obese, or older women. Educating women on healthy habits, like maintaining a healthy weight and being active, can prevent GDM.

GDM is a debilitating disease that affects maternal and prenatal health, and worldwide an alarming 537 million people suffer from T2DM [16,17]. GDM detection is simple, and its early detection and management are known to decrease side effects for the mother and fetus [18]. Several studies have shown that biomarkers, antenatal diet modifications, or early treatment can have beneficial effects on the mother and child [19,20]. Additionally, biomarkers that can be detected before the onset of the disease are helpful as they can help in early intervention [21,22].

One study in a large cohort defined that macrosomia is not only known to cause GDM, but macrosomia in normal women can also lead to T2DM [23]. Although our study did not check whether macrosomia causes T2DM, it clearly showed that GDM can lead to macrosomia (p = 0.020 between GDM and control). Similarly, GDM is known to increase miscarriages, as one recent meta-analysis found that women suffering from GDM have a 41% higher incidence of miscarriages [24]. However, our study did not show any significant differences between control and women suffering from GDM, and this may be due to the homogenous population of Saudi women and the lesser number of participants. On the other hand, our study clearly identified that the prevalence of GDM, a family history of T2DM, earlier, and macrosomia are the risk factors for the development of GDM in Saudi women. One study by Alphadhli et al. found a similar prevalence of GDM (39.4% vs. 42%) in Saudi women [25]. This study assessed risk factors and pregnancy outcomes according to the IADPSG and found that Saudi women were more prone to GDM, and, thus, the prevalence was high. Concurrent with our findings, this study also reported that high blood pressure (p-value = 0.001, control vs. GDM), BMI (p-value = 0.001, control vs. GDM), previous history of GDM (p-value = 0.001, control vs. GDM), and history of birthing malformed children (p-value = 0.027) along with the family history of diabetes (p-value = 0.002) were known risk factors for GDM [25]. However, another study on 15,000 women found a lower prevalence of GDM (24% vs. 42%) [26]. This may be due to the fact that in the former study, data on the glycemic index of a large number of participants were missing, and the population was a mixture of Saudi-born women and expatriates. In agreement with our study, the risk factors found in both studies were similar to ours [25,26]. Several other factors may contribute to the higher prevalence of GDM in Saudi women, such as older age and metabolic disorders during conception [25,27].

In a similar line of thought, a retrospective study by Han et al. of 15,668 Chinese women with no history of diabetes established a linear relationship between GDM risk and age. Age was an important factor, and the risk increased by 8% for every increase of one year of maternal age [28]. This is in concurrence with our study results, which also found that increasing age significantly contributes to GDM. Another study also showed that obesity increases the risk of GDM in women 35 years or more by twofold [29]. Similarly, a retrospective case-control study found that the prevalence of GDM was 15.69% in Chinese women and was 2.117 times higher when the maternal age was more than 35 years. Interestingly, this study also found that the chances of GDM increase by five times if the fathers had diabetes and pointed to the fact that the number of pregnancies increases the chances of GDM and miscarriage [30]. Several other studies have identified smoking as a known factor that increases the chances of GDM, but we did not find any association of GDM with smoking in our study [31,32]. This may be attributed to the large number of study participants enrolled in these studies.

Our study sheds light on a topic of relevance, as diabetes in pregnant women is detrimental to their overall health and may lead to chronic diabetes after birth. In brief, this study has broader applications and can be applied to a larger population. In our study, there is a chance of inaccuracies or inherent bias as the data was collected through pre-defined forms. Moreover, our study was only for one year, and long-term follow-up is required to decipher factors that cause GDM in mothers and children. Additionally, there may be other genetic and epigenetic factors that our study did not explore. Data collection was limited by time. For example, information on pre-pregnancy BMI, dietary intake, exercise during pregnancy, and long-term prognosis after childbirth could not be accurately obtained. Additionally, since the study is record-based information, missing data is found as previous miscarriages, smoking status, family history, occupation, and education level.

## Conclusions

The results of this study suggest that several factors are associated with an increased risk of developing GDM, including a family history of diabetes, a history of GDM, and macrosomia. The study also found that overweight/obesity and advanced maternal age were risk factors for GDM, which is consistent with previous research. However, the study did not find a significant association between GDM and other factors, such as chronic disease prevalence, history of miscarriage, or history of fetal death. It is recommended that healthcare providers in antenatal care clinics screen pregnant women for risk factors associated with GDM, such as a family history of diabetes and a history of GDM. Additionally, women who are overweight or obese or of advanced maternal age should be closely monitored for the development of GDM. Education and counseling on healthy lifestyle habits, such as maintaining a healthy weight and engaging in physical activity, may also be beneficial for preventing GDM.

Further research is needed to explore and identify additional risk factors for GDM. Additionally, future studies may benefit from exploring interventions for managing GDM and its associated risks, such as early screening and treatment, patient education and counseling, and improved monitoring and follow-up care. Further research may also explore the effectiveness of lifestyle interventions, such as diet and exercise, in preventing and managing GDM.
